# Physical Exercise Therapy effect on Cholesterol Profile of Youth at Mental Health Secure Inpatient Service

**DOI:** 10.1192/j.eurpsy.2026.11123

**Published:** 2026-07-31

**Authors:** C. Schneider

**Affiliations:** Sport psychiatry, Royal College of Psychiatry, London, United Kingdom

## Abstract

**Introduction:**

Physical activity was found to be associated with an increase in HDL cholesterol (HDL-C), and a decrease in LDL cholesterol and triglycerides in adults. Studies have shown that exercise reduces the risk of cardiovascular disease partially through increasing serum levels of HDL-C. In parallel, low HDL-C levels are associated with a higher risk of developing depression and other psychiatric disorders. High levels of HDL- C is known to be a marker of good cardiovascular health, while low HDL is a predictor of heart disease. The relationship between HDL- C and mental disorders is complex and may involve shared mechanisms such as inflammation and metabolic changes. The effect of high levels of HDL-C can have in young people’s physical and mental health has been less explored.

**Objectives:**

To evaluate the effect of Physical Exercise Therapy on HDL-C levels in young people admitted to a secure inpatient setting.

**Methods:**

Data of physical health parameters (cholesterol levels, weight, full blood count) of 19 adolescents admitted to a secure inpatient service between December 2022 and March 2025, who engaged in physical exercise therapy sessions collected and analyzed. Data was collected on admission, three months after participating in Physical Exercise Therapy and on discharge.

**Results:**

We found that Yong People who participated in regular physical exercise as part of a planned care programme showed a significant increase in levels of HDL-C.

**Conclusions:**

Physical Exercise Therapy showed to increase the levels of HDL-Cholesterol. The increase of HDL-C levels in young people admitted to inpatient settings not only has a positive impact in reducing the risk of metabolic syndrome and heart disease, but also might improve young people’s mental state and overall functioning, reducing the risk of potential serious outcome related to their psychopathology. Further studies are needed

**Disclosure of Interest:**

None Declared

